# The Organization of the Pig T-Cell Receptor γ (TRG) Locus Provides Insights into the Evolutionary Patterns of the *TRG* Genes across Cetartiodactyla

**DOI:** 10.3390/genes13020177

**Published:** 2022-01-19

**Authors:** Giovanna Linguiti, Francesco Giannico, Pietro D’Addabbo, Angela Pala, Anna Caputi Jambrenghi, Salvatrice Ciccarese, Serafina Massari, Rachele Antonacci

**Affiliations:** 1 Department of Biology, University of Bari “Aldo Moro”, Via E. Orabona 4, 70125 Bari, Italy; giovanna.linguiti@uniba.it (G.L.); pietro.daddabbo@uniba.it (P.D.); angela.pala@uniba.it (A.P.); salvatricemaria.ciccarese@uniba.it (S.C.); 2 Department of Veterinary Medicine, University of Bari “Aldo Moro”, Strada Provincial 62 per Casamassima Km 3, 70010 Bari, Italy; francesco.giannico@uniba.it; 3 Department of Agricultural and Environmental Science, University of Bari “Aldo Moro”, Via E. Orabona 4, 70125 Bari, Italy; anna.caputijambrenghi@uniba.it; 4 Department of Biological and Environmental Science and Technologies, University of Salento, 73100 Lecce, Italy; sara.massari@unisalento.it

**Keywords:** pig genome, γ/δ T-cell, TRG locus, *TRG* genes, γ/δ high species, Cetartiodactyla, immunogenomics, evolution

## Abstract

The domestic pig (*Sus scrofa*) is a species representative of the Suina, one of the four suborders within Cetartiodactyla. In this paper, we reported our analysis of the pig TRG locus in comparison with the loci of species representative of the Ruminantia, Tylopoda, and Cetacea suborders. The pig TRG genomic structure reiterates the peculiarity of the organization of Cetartiodactyla loci in TRGC “cassettes”, each containing the basic V-J-J-C unit. Eighteen genes arranged in four TRGC cassettes, form the pig TRG locus. All the functional *TRG* genes were expressed, and the *TRGV* genes preferentially rearrange with the *TRGJ* genes within their own cassette, which correlates the diversity of the γ-chain repertoire with the number of cassettes. Among them, the *TRGC5*, located at the 5′ end of the locus, is the only cassette that retains a marked homology with the corresponding TRGC cassettes of all the analyzed species. The preservation of the TRGC5 cassette for such a long evolutionary time presumes a highly specialized function of its genes, which could be essential for the survival of species. Therefore, the maintenance of this cassette in pigs confirms that it is the most evolutionarily ancient within Cetartiodactyla, and it has undergone a process of duplication to give rise to the other TRGC cassettes in the different artiodactyl species in a lineage-specific manner.

## 1. Introduction

Artiodactyla represents the largest order of terrestrial mammals. In the past, Artiodactyla was considered monophyletic and was traditionally divided into three major lineages: Suina, Tylopoda, and Ruminantia [1]. The modern phylogeny, based on molecular and paleontological data [2,3,4], recognized that Cetacea, a highly specialized mammalian order that includes whales, dolphins, and porpoises, is a nested member of Artiodactyla. Therefore, Cetartiodactyla has become the generally accepted name for the superorder containing both orders, and it currently includes over 330 species grouped into 23 families and 131 genera [5].

However, the phylogeny of this superorder is controversial regarding the relationships among the major clades of cetartiodactyls. Different studies indicate a certain instability at the root of the phylogeny, varying between Suina or Tylopoda in stating the first lineage to diverge [4,6,7,8,9,10,11], or, alternatively, postulating a monophyletic clade containing Tylopoda and Suina as the sister group to Cetacea and Ruminantia [6,12].

Comparative phylogenomic studies, coupled with the development of genomic assemblies for the species belonging to this superorder, can help to resolve the controversies.

Among genomic regions, the complex loci encoding for the T-cell receptor (TR) chains provide valuable resources for the analysis of specific traits and comparative studies in the various species of Cetartiodactyla.

Two separate lineages, characterized as either or T lymphocytes according to the expression of TR on their membranes, comprise the total T-cell pool in mammals. In contrast to αβ TR, which only recognize antigens as peptide fragments bound to major histocompatibility complex (MHC) molecules, a feature known as MHC restriction, γδ T cells recognize antigens in their native forms and are not MHC-restricted. The γδ T cells were initially identified in humans and mice, and no obvious conservation of γδ T-cell subsets, based on TR repertoire and function, between the two species, was found, leading to the notion that human and mouse γδ T cells are highly different [13]. γδ T cells have also been studied in animals other than humans and mice.

In artiodactyl species, γδ T cells represent a set of the T population whose characteristics are diverse, and, as in humans and mice, their functions in these species have not yet been well defined [13]. For example, they comprise up to 60% of the circulating lymphocytes in young cattle, sheep, and swine, and, although the percentage decreases with age, they still represent up to 30% in adults. γδ T cells also account for up to 35% of the blood lymphocytes in newborn and young camels, and although the proportion is lower than that in other artiodactyl species, they are still a major population in the blood [14]. Therefore, ruminants and pigs, as well as camels, are γδ T-cell “high-species” with respect to humans and mice, which are defined as γδ T “low-species” because of the low percentage (approximately 5%) of γδ T cells in the peripheral blood [13].

Moreover, the potential diversity of the γ and δ chain repertoires is wider and more diverse in the artiodactyl species compared to the restricted and limited repertoire in humans and mice. In turn, the γ and δ chain repertoires depend on the gene organization, which interestingly shows high variability among different species [13,15,16,17,18].

The γ and δ chains are encoded by separate multigene families. Each chain is generated by somatic rearrangements of non-contiguous germline genes belonging to the variable (*V*), diversity (*D*; only for the δ chain), and joining (*J*) gene types organized in complex TR genomic loci that lie, for each chain, in specific chromosomal regions. During recombination, one of the multiple *V*, *D*, and *J* genes is selected and they are joined together to form the variable portion of the chain. After transcription, the *V*-(*D*)-*J* sequence is spliced to the constant (*C*) gene encoding for the constant portion of the chain. The random insertion and deletion of nucleotides at the rearrangement positions create junctional diversity in the highly variable complementarity determining region 3 (CDR3), which is primarily responsible for antigen recognition. Two other, more variable regions (CDR1 and CDR2) are encoded by the germline *V* genes.

The genomic organization of the locus encoding for the TR γ chain (TRG) is the most considerably different across vertebrates and seems to be related to the evolution of species [13,17].

In cetartiodactyl species (sheep, goats, cattle, dromedaries, and dolphins), the genomic structure of the TRG locus is characterized by the presence of a basic structural scheme, consisting of a V-J-J-C unit or “cassette” [13]. The basic unit also includes, at the 5′ end of the first *J* gene, a promoter for the germline transcription, and at the 3′ end of the *C* gene, an enhancer-like element, which controls the local recombinational accessibility.

The number of cassettes varies in the different species as a consequence of reiterated duplications, with a single cassette in dolphins, three in dromedaries, and six (sheep), or seven (goats and cattle) in ruminants, arranged in the last in two distinct TRG loci that map separately on the same chromosome [13].

In this study, we investigated the genomic organization of the TRG locus in *Sus scrofa*, a species that we considered representative of the Suina suborder, to understand the origin and evolutionary dynamics of the TRG loci in the cetartiodactyl group. Our comparative data provide new insights into the evolutionary patterns of the *TRG* genes within Cetartiodactyla, but also the implications for clarifying the phylogenetic relationships among the major clades of this important group of mammals.

Moreover, an expression assay on the spleen RNA sample revealed that all the annotated functional *TRG* genes contributed to the pig γ chain repertoire. Each *TRGV* gene preferentially rearranges with the *TRGJ* genes of its own cassette, and the *V*-*J* region is spliced to the relevant *TRGC* in the mature transcripts. Therefore, the number of cassettes seems to be relevant for the level of diversity of the γ chain repertoire.

## 2. Materials and Methods

### 2.1. Pig Genome Analysis

To determine the pig TRG locus location, the Sscrofa11.1 genome sequence (GenBank accession: GCA_000003025.6), released by the International Swine Genome Sequencing Consortium, was searched using the BLAST algorithm. A sequence of 317,500 bp was retrieved directly from the reference sequence NC_010451.4 (*Sus scrofa* chromosome 9 genomic sequence) available at NCBI from 108,654,001 to 108,971,498 (complement) positions. Particularly, the analyzed region extended from the *AMPH* to the *STARD3NL* genes found flanking the TRG locus of most mammalian species (https://www.imgt.org/IMGTrepertoire/LocusGenes/#h1_6, accessed on 20 December 2021).

All *TRG* genes within the genome sequence were identified and annotated using the available pig TRG cDNA collection (Acc. numbers: AB185441-AB185447) and other artiodactyl TRG genomic sequences as a reference. The beginning and end of each coding exon were identified with accuracy by the presence of splice sites or flanking recombination signal (RS) sequences of the *V* and *J* genes.

The locations of the *TRG* genes are provided in Supplementary Table S1 together with the position of *AMPH* and *STARD3NL* genes.

Moreover, computational analysis of the pig TRG locus was conducted using the RepeatMasker for the identification of genome-wide repeats and low complexity regions (Smit, A.F.A., Hubley, R., Green, P. RepeatMasker open-4.0. at http://www.repeatmasker.org, accessed on 20 December 2021) and Pipmaker [19] for the alignment of the determined pig sequence with itself. The inspection of the obtained dot-plot matrix allowed us to identify portions of the sequence that align with more regions within the sequence itself.

For the identification of the regulatory regions in the pig TRG locus, the nucleotide sequences of 598 bp and 771 bp, corresponding, respectively, to the human 5′ J γ promoter (PJ) and 3′ enhancer element (En), were retrieved from the human TRG genomic sequence (Accession no. NC_01045: pos. 38276916-38276319 for PJ and pos. 38233337-38232567 for En) and compared with the pig corresponding sequences by Clustal Omega [20] at www.ebi.ac.uk (accessed on 20 December 2021).

For the genomic analysis of the Cetoartiodactyla TRGC cassettes, each sequence, which comprises the first *TRGV* gene until the enhancer-like element, was retrieved from each TRG locus of the different species. For simplicity, we considered the cassette sequences derived from a single representative species for each suborder of Cetartiodactyla, i.e., sheep for Ruminantia, pig for Suina, dromedary for Tylopoda, and dolphin for Cetacea. The position of the *TRG* genes was annotated in Genbank format by the SnapGene Viewer utility (SnapGene Viewer 5.3.2, available at www.snapgene.com accessed on 20 December 2021). The annotated sequences were compared to each other by Mauve, a genome multiple alignment software (Mauve 2.4.0, available at darlinglab.org/mauve/mauve.html, accessed on 20 December 2021).

### 2.2. Classification of the Pig TRG Genes

The functionality of the *V*, *J*, and *C* genes was predicted through the manual alignment of sequences adopting the following parameters: (a) identification of the leader sequence at the 5′ of the *V* genes; (b) determination of proper RS sequences located at 3′ of the *V* (V–RS) and 5′ of the *J* (J-RS), respectively; (c) determination of conserved acceptor and donor splicing sites; (d) estimation of the expected length of the coding regions; (e) absence of frameshifts and stop codons in the coding regions of the genes. Conversely, a germline gene is qualified as ORF (open reading frame) if the coding region has an open reading frame, but alterations have been described in the splicing sites and/or RS sequences, and/or in changes of conserved amino acids. Finally, a germline gene is qualified as a pseudogene (P) if its coding region has stop codon(s) and/or frameshift mutation(s). According to the germline sequences, four *TRGV* genes are pseudogenes: *TRGV4*, *TRGV6*, *TRGV11*, and *TRGV12-2*. However, we have considered the *TRGV6* (with frameshift in L-PART1 and non-canonical donor splice site) and *TRGV12-2* (with frameshift in V-exon) functional genes and modified their nucleotide sequence based on the sequence of productive cDNA clones (GenBank Accession N° AB185445 and AB185447) that retain the two genes (Supplementary Table S1).

The *TRGV* genes were grouped in different subgroups based on the percentage of nucleotide identity by using the Clustal Omega alignment tool, which is available at the EMBL-EBI website (http://www.ebi.ac.uk/, accessed on 6 May 2021), adopting the criterion that sequences with a nucleotide identity of more than 75% in the coding region of a TR *V* gene (i.e., L-PART1+V-EXON) belong to the same subgroup [21]. 

The *TRGJ* genes were named by a number in accordance with the name of the belonging TRGC cassette, followed by a hyphen and a number corresponding to their position within the cassette.

The *TRGC* genes, classified on the basis of the homology with the ruminant corresponding genes, define the name of the cassettes. All pig *TRGC* genes were predicted to be functional, even the *TRGC4* gene, whose EX1 was out of frame in the assembly probably due to a sequence error (Supplementary Table S1). In fact, the sequence of the first exon of the pig *TRGC4* gene lacked a “c” nucleotide in position 114, which was present in the same gene sequence detected in a previous genomic assembly (Sscrofa10.2) and in a cDNA clone (GenBank accession no. L21161).

### 2.3. Phylogenetic Analysis

The human, bovine, sheep, dolphin *TRGV*, and *TRGC* gene sequences used for the phylogenetic analysis, as annotated, were retrieved from the IMGT^®^ database (IMGT Repertoire, http://www.imgt.org, accessed on 20 December 2021), IMGT/GENE-DB, [22]. The goat, dromedary, and pig gene sequences were retrieved from the GenBank database with the following accession numbers: NC_030811.1 (goat TRG locus contig as characterized by Giannico et al. [17]); GCA_000803125.1, JN165102, and JN172913 (dromedary TRG locus as characterized by Antonacci et al. [23]); and NC_010451.4 (pig TRG locus as characterized in this work). We combined the nucleotide sequences of the V-REGION of the pig *TRGV* genes with the corresponding gene sequences of sheep, goats, cattle, dromedaries, dolphins, and humans. Similarly, the pig *TRGC* gene nucleotide sequences, as well as their 3′UTR nucleotide sequences (from the stop codon to the poly-A site), were aligned with the corresponding ruminant, dromedary, and dolphin gene sequences.

Multiple alignments of the gene sequences under analysis were carried out with the MUSCLE program [24]. The evolutionary analyses were conducted in MEGA X [25,26]. We used the neighbor-joining (NJ) method to reconstruct the phylogenetic tree [27]. The evolutionary distances were computed using the p-distance method [28] and are in the units of the number of base differences per site.

Based on the TRGC phylogenetic tree, the RelTime method [29,30] was employed to compute the mean substitution rates along each branch and estimate the species divergence time. The chicken *TRGC* gene sequence was specified as the outgroup. The TimeTree was computed using one constraint to estimate absolute times. We choose a time range of 66 to 68 Ma, which represents the occurrence of divergence between dromedary (Tylopoda) and pig (Suina), as the calibration point. The time range was derived from previous estimations based on fossil records and molecular data [11].

### 2.4. 5′ Rapid Amplification of cDNA Ends (RACE) PCR

Total RNA was extracted from the spleen of an adult animal using the Trizol method according to the manufacturer’s protocol (Thermo Fisher Scientific, Waltham, MA, USA). Approximately 5 ug of RNA was reverse transcribed with Superscript II (Thermo Fisher Scientific) by using an oligo-dT adapter primer.

After linking a poly-C tail at the 5′ end of the ss cDNA, two different amplification experiments were set up to perform the ds cDNA with Platinum Taq polymerase (Thermo Fisher Scientific). In one PCR reaction, GL1L (5′ TCCAGAAGACAAAGGTATGTTCCA 3′) was used as the lower primer, which was designed on a conserved nucleotide sequence of the first exon shared by the pig *TRGC3*, *TRGC4*, and *TRGC6* genes. The second PCR experiment was performed using the lower primer GL1Lbis (5′ TCAAGAAGACAAAGATGTGTCCCA 3′), designed on the sequence of the first exon of the pig *TRBC5* gene. In both PCR reactions, an anchor oligonucleotide was used as the upper primer (AAP) provided by the supplier (Thermo Fisher Scientific). The PCR conditions were as follows: 30 s at 94 °C, 45 s at 55 °C, and 1 min at 72 °C for 35 cycles. The products were then amplified in a subsequent nested PCR experiment by using the GL2L/AUAP primer pair. GL2L (5′ TATYTCAGCAATYGAAGGAAG 3′, where Y is A or G) was designed on a sequence upstream of GL1L as well as GL1Lbis, while the AUAP oligonucleotide was provided by the supplier (Invitrogen). The PCR conditions were as follows: 15 s at 94 °C, 15 s at 60 °C, 15 s at 72 °C for 30 cycles. The final cycle was extended for 30 min at 72 °C.

The RACE products were then gel-purified and cloned using the StrataClone PCR Cloning Kit (Stratagene). Random selected positive clones for each cloning were sequenced by a commercial service. cDNA sequence data were processed and analyzed using the Blast program (http://www.blast.ncbi.nlm.nih.gov/Blast.cgi, accessed on 20 December 2021), the Clustal Omega alignment tool (http://www.ebi.ac.uk/, accessed on 20 December 2021), and IMGT tools [IMGT/V-QUEST [31,32] with integrated IMGT/Junction Analysis tools [33,34] and the IMGT unique numbering for the V domain [35] (http://www.imgt.org/, accessed on 20 December 2021).

All cDNA clones were registered in the EMBL database with the Accession numbers from OL906430 to OL906434.

## 3. Results

### 3.1. Genomic Structure of the TRG Locus in Sus Scrofa

The public genomic assembly Sscrofa11.1 was analyzed in order to isolate the TRG locus in pigs, a species that was considered as the reference for the Suina suborder of Artiodactyla in our subsequent comparative analyses. To this end, the pig TRG locus was characterized and annotated according to the rules of the international IMGT database (IMGT^®^, http://www.imgt.org, accessed on 20 December 2021).

**Figure 1:**
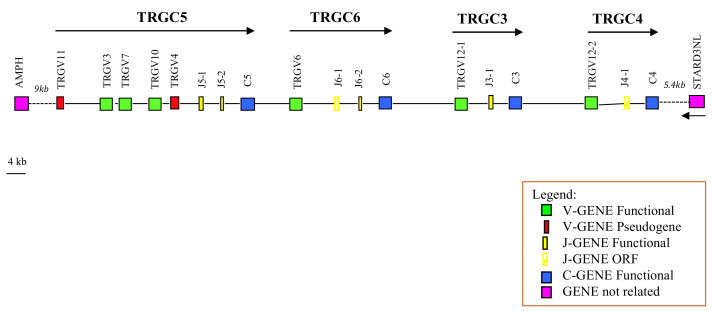
Schematic representation of the genomic organization of the pig TRG locus deduced from the genome assembly Sscrofa11.1. The name of each TRGC cassette is indicated by an arrow. The *TRGV6* and *TRGV12-2* genes, whose coding sequences are not in frame within the assembly, are indicated in the map as functional since they were found in productive cDNA clones (see text). The diagram shows the position of all related and unrelated *TRG* genes according to nomenclature. The boxes representing the genes are not to scale. The exons are not shown. The arrow indicates the transcriptional orientation of the *STARD3NL* gene.

A sequence of approximately 317 kb was recovered. The *TRG* genes occupied about 113 kb and were flanked by the AMPH and STARD3NL genes at the 5′ and 3′ ends, respectively (Figure 1).

Basically, the deduced genomic structure of the TRG locus in pigs reflected the peculiarity of the organization of this region in the other artiodactyl species, consisting of a set of closely related “cassettes”, each containing the V-J-J-C basic unit arranged in the same transcriptional orientation [13,17]. Particularly, the pig TRG locus comprised eight *TRGV*, six *TRGJ*, and four *TRGC* genes distributed in four V-J-(J)-C cassettes, which was fewer than the number of cassettes characterizing the ruminant TRG locus (seven in cattle and goats, and six in sheep) [13,17], but it was greater than the number present in the dromedary (three cassettes) [23] and dolphin (a single cassette) [36] loci. The total number of *TRG* genes in pigs was also lower than that of ruminants (36 in goats, 34 in cattle, and 32 in sheep), but comparable to that in dromedaries (17 genes) and higher than that in dolphins (six genes). The pig cassettes were classified, proceeding from the 5′ to the 3′ end of the region, as *TRGC5*, *TRGC6*, *TRGC3*, and *TRGC4*, in accordance with the corresponding ruminants’ cassettes (see the paragraph below for details; IMGT^®^, http://www.imgt.org, accessed on 20 December 2021). Five *TRGV* genes were in the *TRGC5* cassette, and only one was in each of the other three cassettes. Two *TRGJ* genes composed the *TRGC5* and *TRGC6* cassettes, while only one *TRGJ* gene was present in the *TRGC3* as well as in the *TRGC4* cassette. Finally, each cassette comprised a *TRGC* gene that was assigned the same name as the cassette.

### 3.2. Gene Analysis

The *TRGV* genes were assigned to seven different subgroups by nucleotide-sequence identity (see Section 2.2). All the subgroups consisted of a single *TRGV* gene except for the TRGV12 subgroup, which consisted of two genes.

Six are predicted to be functional genes as defined by the IMGT rules (see Section 2; IMGT^®^, http://www.imgt.org, accessed on 20 December 2021), and only two are pseudogenes (Supplementary Table S1). The structure of the pig germline *TRGV* genes is shown in Supplementary Figure S1A.

**Figure 2:**
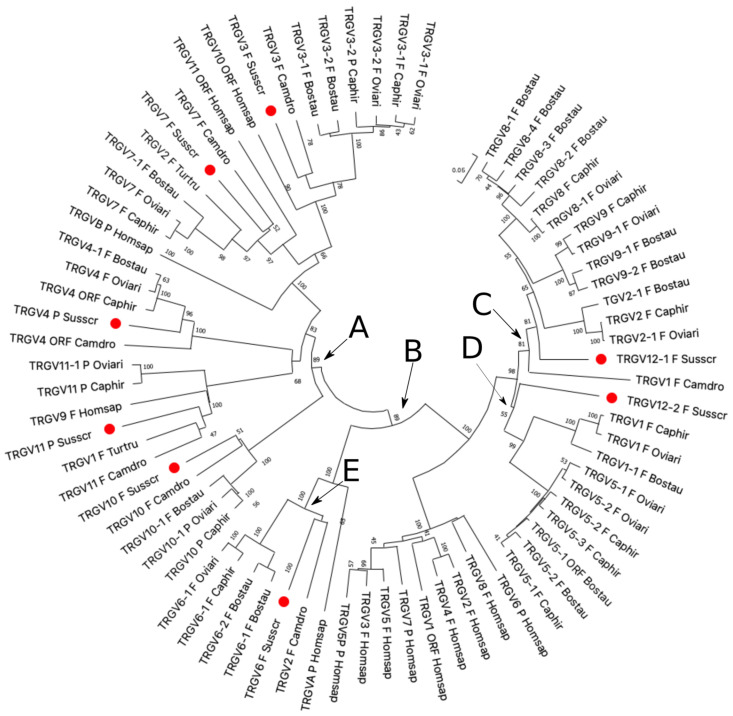
The neighbor-joining (NJ) tree inferred from the pig, sheep, goat, bovine, dromedary, dolphin, and human TRGV gene sequences. The evolutionary analyses were conducted in MEGA X [25,26]. The optimal tree with the sum of branch length = 5.56210337 is shown. The percentage of replicate trees in which the associated taxa clustered together in the bootstrap test (100 replicates) is shown next to the branches [37]. The tree is drawn to scale, with branch lengths in the same units as those of the evolutionary distances used to infer the phylogenetic tree. The evolutionary distances were computed using the p-distance method [28] and are in the units of the number of base differences per site. This analysis involved 45 nucleotide sequences. Codon positions included were 1st + 2nd + 3rd + Noncoding. All ambiguous positions were removed for each sequence pair (pairwise deletion option). There was a total of 349 positions in the final dataset. Each pig *TRGV* gene is marked with a red circle. The branches highlighted by the letters group mammalian genes described in the text. The gene functionality according to IMGT rules (F: functional, ORF: open reading frame, P: pseudogene) is indicated. The IMGT 6-letter for species (Susscr, Bostau, Oviari, Caphir, Camdro, Turtru, and Homsap) standardized abbreviation for a taxon was used.

The classification of the pig *TRGV* genes to the subgroups was established by phylogenetic analysis in the context of the Cetartiodactyla superorder by comparing all the pig gene sequences with the corresponding ruminant (goat, sheep, and cattle), dromedary, and dolphin gene sequences. The human genes were also included in the analysis. Thus, the V-REGION nucleotide sequences of all the selected *TRGV* genes were combined in the same alignment, and an unrooted phylogenetic tree was constructed using the NJ method [27] (Figure 2).

The tree shows that each pig TRGV subgroup formed a monophyletic group with corresponding ruminant genes and, when present, with corresponding genes of dromedaries, dolphins, and humans, consistent with the birth of each pig subgroup from a mammalian common ancestor prior to the divergence of the different species. Two major groupings of the mammalian genes were clearly distinguishable in the tree. ranch A grouped genes exhibiting a conserved nature across species, with a clear orthology. This branch included all the *TRGV* genes belonging to the artiodactyl TRGC5 cassette [13]. Therefore, each pig TRGV gene subgroup was named in accordance with each corresponding artiodactyl gene subgroup name, i.e., TRGV3, TRGV4, TRGV7, TRGV10, and TRGV11. The artiodactyl *TRGC5* cassette has been shown to be the most evolutionarily ancient [13]. It would have been duplicated to generate a second one that developed the other artiodactyl TRGC cassettes, whose *TRGV* genes are all included in branch B, where they formed three principal groupings, C, D, and E (Figure 2). In C, all the ruminant genes belonging to distinct subgroups (TRGV2, TRGV8, and TRGV9) and located in the *TRGC3* (named *TRGC7* in cattle) cassette, were clustered. Only one pig *TRGV* gene and the dromedary *TRGV1* gene were closely related to these genes as paraphyletic branches. Hence, in the absence of a perfect (univocal) orthology, we classified the pig gene as belonging to a new subgroup. It was named *TRGV12-1* since the pig TRGV12 subgroup consisted of two genes. The pig *TRGV12-2* gene groups with paralogous genes located in the ruminant *TRGC4*, *TRGC1*, and *TRGC2* cassettes (D branch). Finally, the E branch grouped the *TRGV* genes belonging to the ruminant TRGV6 subgroup and located in the TRGC6 cassette together with the orthologous pig *TRGV6* and the dromedary *TRGV2* genes. In accordance with these phylogenetic data, we classified the pig *TRGC* cassettes, proceeding from the 5′ to the 3′ end of the region, as the ruminant *TRGC5*, *TRGC6*, *TRGC3*, and *TRGC4*.

Supplementary Figure S1B reports the nucleotide and deduced amino-acid sequences of all the pig *TRGJ* genes identified in the region. The genes were 49–60 bp long and show conservation of the canonical FGXG(A) amino-acid motif, whose presence defines the functionality of *J* genes. The only exception was the *TRGJ4-1* gene, which had a different amino acid in the second position of the motif and was therefore classified as ORF (Supplementary Table S1). Notably, *TRGJ4-1* was found to be rearranged within one cDNA clone (GenBank accession no. AB185447). Each *TRGJ* gene was flanked by a 12 RS at the 5′ end and by a donor splice site at the 3′ end. The RS were well conserved in the crucial positions with respect to the consensus sequence, except for the first and second nucleotide of the “cac” sequence of the heptamer of the *TRGJ6-1* gene that was classified as ORF.

The structure of the pig *TRGC* genes was similar to that of the homologous mammalian genes (http://www.imgt.org/IMGTrepertoire/Proteins/; accessed on 20 December 2021) (Supplementary Figure S1C). The first exon (EX1) encoded the C domain, while the first part of the connecting region was encoded by one (EX2A) or three (EX2A, EX2B, and EX2C) exons. The remaining portion of the connecting region, the transmembrane region, and the cytoplasmic region were encoded by EX3. Hence, the connecting region of the pig *TRGC* genes differed in length and amino-acid sequence depending on the encoding of EX2 exons. This heterogeneity is shared with the *TRGC* genes of other artiodactyl species, where the connecting region can be encoded by three exons, two exons, or only one exon [13]. In particular, the pig *TRGC5* consists of only one EX2, as observed in ruminants, dromedaries, and dolphins. Moreover, in pigs as in camels, the *TRGC* gene consisting of two EX2 is missing compared to that in ruminants.

### 3.3. 5′ RACE Assay

To evaluate the features of the expressed γ chain repertoire with respect to the germline genes identified in the genome assembly, we performed a 5′ RACE assay on an RNA sample isolated from the spleen of an adult animal. After the reverse transcription, two different amplification experiments were set up. In the first, we used GL1L as a lower primer designed based on a conserved sequence of the first exon shared by the *TRGC3*, *TRGC4*, and *TRGC6* genes. In the second, the lower primer GL1Lbis, designed on the first exon of the *TRGC5* gene, was used. The two amplification products were separately cloned, and 35 randomly selected positive clones for each cloning were sequenced. Sequence analysis revealed that all the clones consisted of rearranged V-J-C transcripts of different lengths, but most of them were redundant cDNAs. Five different transcripts were obtained. Four cDNAs (GL2L6, GL2L13, GL2L17, and GL2L36) were isolated from the first amplification experiment and one clone (GL1L2) from the second one (Supplementary Figure S2).

Each sequence was manually analyzed to identify the *TRGV*, *TRGJ*, and *TRGC* genes through alignment with the germline pig genes annotated in the assembly. Supplementary Figure S2 shows the protein display of these cDNA sequences together with further cDNA clones from pig spleen retrieved from the IMGT database (accession no. AB185441-AB185447). The comparison of the expressed sequences with the germline ones allowed us to ascertain that all the cDNA perfectly matched with the corresponding germline TRGV, TRGJ, and TRGC sequences, with the exception of a single mismatch in the TRGV6 gene sequences, which resulted in an amino-acid change (T instead of N) in cDNAs (Supplementary Figure S2). In spite of the small collection of cDNA clones, we can say that all the annotated *TRGV* genes but one (*TRGV11* pseudogene) are used in the V-J somatic recombination for the γ chain. Even the *TRGV4* gene, classified as an in-frame pseudogene, is included. Likewise, all the *TRGJ* genes are used except for the *TRGJ6-1* gene, which presents no canonical RS sequence, accounting for the lack of transcription.

Finally, the sequence analysis of the constant portion in each cDNA revealed the presence of the *TRGC* gene proximal to the *TRGJ* gene used in the V-J rearrangement.

Therefore, the sequence analysis of the cDNAs confirmed, as in other artiodactyl TRG loci, that each *TRGV* gene preferentially rearranges with the *TRGJ* genes of its own cassette, and the V-J region is spliced to the relevant *TRGC* in the mature transcripts [38,39], leading to the conclusion that the cassette number is crucial for the diversity of the γ chain repertoire. However, the number of cassettes in pigs seems to limit the potential gene recombinations compared to other artiodactyls. Indeed, in ruminants, the greater number of *TRG* genes distributed within a greater number of reiterated cassettes increases the potential rearrangements [13,17]. The level of diversity of the pig γ chain repertoire was lower not only than that of ruminants but also than that of dromedaries. As a matter of a fact, recent studies [23,40,41] have provided evidence that the somatic hypermutation mechanism (SHM) contributes to the expansion of the diversity of the dromedary γTR repertoire, even though only three cassettes lie in the locus.

However, a recent deep expression study [39] performed in different pig lymphoid tissues and T-cell populations confirmed that about 75% of the rearrangements were within a cassette, but there was also trans-cassette V-J recombination and VJ-C trans-cassette splicing. Curiously, the last two mechanisms do not seem to occur for the TRGC5 cassette, where most of the *TRGV* genes are located.

### 3.4. Genomic Architecture and Identification of the Regulatory Elements in the Pig TRG Cassettes

**Figure 3:**
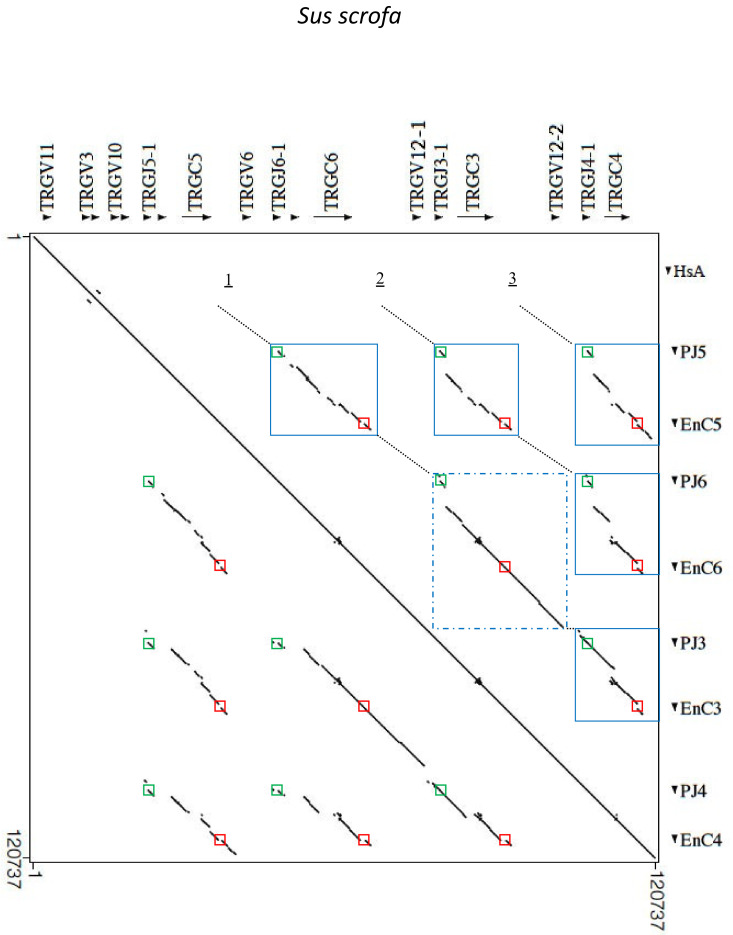
Dot-plot of the pig TRG locus sequence against itself. The transcription orientation of each sequence is indicated by arrows and arrowheads. Three parallel lines (1–3) to the perfect main diagonal line indicate the internal homology units between the TRGC cassettes. The interruptions of the parallel lines are indicated by dotted lines. The blue boxes show the internal homology of the J-C gene blocks. The blue dashed square indicates the longest duplicated region containing the TRGJ6-1/TRGC6/TRGV12-1/TRGJ3-1/TRGC3/TRGV12-2 gene block.

The genomic architecture of the pig TRG locus was further investigated, aligning the masked corresponding sequence (from the *TRGV11* to *TRGC4* gene) against itself with the Pipmaker program (Figure 3). The dot-plot matrix detected the occurrence of internal homology units along the entire pig TRG region, as indicated by dots and lines. Parallel lines clearly showed the mode of evolution of the locus by tandem duplications of V-J-J-C cassettes. Three lines parallel to the perfect main diagonal line, which indicated the match of each base of the sequence with itself, highlighted the high level of nucleotide identity between the four pig TRGC cassettes. Each parallel line was interrupted due to the lack of nucleotide identity between the TRGV-gene-containing regions of the different cassettes. Therefore, units of internal homology were detected just between the J-C gene blocks (the blue squares in Figure 3). The only exception was the clear homology between the TRGV12 subgroup genes, which resulted in the longest homology unit consisting of a TRGJ6-1/TRGC6/TRGV12-1/TRGJ3-1/TRGC3/TRGV12-2 gene block (blue dashed square in Figure 3). The high homology between the TRGC3 and TRGC4 gene cassettes, even within their TRGV region, supports the idea that they may arise from a recent duplication event.

It should be noted that the lines of similarity corresponding to each J-C gene block extended beyond the TRGJ and TRGC coding regions, comprising the germline transcription promoters (PJ) located at the 5′ end of each *TRGJ* gene distal to the relevant *TRGC* gene (green boxes in Figure 3), and the enhancer-like elements (En) positioned about 2 kb from the 3′ ends of all the *TRGC* genes (red box in Figure 3), respectively. These regions have been experimentally determined in humans [42,43] as the cis-control elements that cooperate to regulate the accessibility of the TRG locus to the site-specific recombination machinery through their possible interaction with STAT5 proteins. A comparison between the human cis-control elements and the corresponding pig sequences revealed high conservation in the pig sequences of 400 bp in the PJ (Supplementary Figure S3A) and 153 bp in the En (Supplementary Figure S3B) regions, where STAT consensus motifs (TTCNNNGAA) were located. Therefore, the functional value of the PJ and En regions is underlined by their conservation, which led to the need for them to duplicate themselves together with the rest of the cassettes.

### 3.5. Phylogenetic Relationships of the Cetoartiodactyla TRGC Genes

The availability of the pig germline sequences allowed us to estimate the timing and mode of TRGC gene duplications along the evolutionary history of cetartiodactyl species. We constructed a TimeTree by aligning the coding nucleotide sequences of the pig *TRGC* genes with the corresponding ruminant (goat, sheep, and cattle), dromedary, and dolphin ones. The chicken *TRGC* gene sequence was used as the outgroup. For the calibration of the phylogenetic tree (evolutionary time), we used the time of divergence between dromedaries and pigs. The tree highlighted that the evolutionary relationship among the *TRGC* genes in the different species was mostly consistent with the current phylogeny. As a matter of a fact, all the sequences, except for the *TRGC5* pig-related genes, made lineage-specific clusters, which showed consistency between the estimated divergence time and the accepted phylogenetic divergence time of each species.

**Figure 4:**
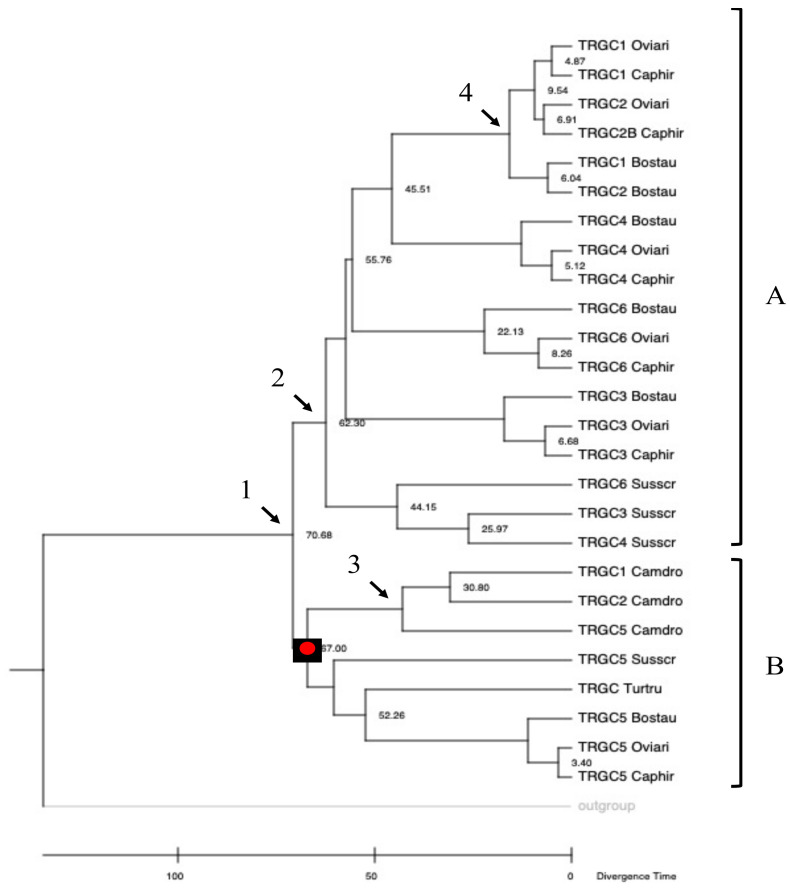
Time-calibrated phylogenetic tree of *TRGC* genes in Cetartiodactyla derived from MEGAX [25] applying the RelTime method. This analysis involved 27 nucleotide sequences. Codon positions included were 1st + 2nd + 3rd + Noncoding. All ambiguous positions were removed for each sequence pair (pairwise deletion option). There were a total of 818 positions in the final dataset. The calibration point (red circle) was arranged on a node to indicate the occurrence of divergence between Tylopoda (Camdro), and Suina (Susscr) estimated on fossil records and molecular data [11]. The chicken TRGC sequence was used as the outgroup. A (ruminant and pig *TRGC* genes) and B (dromedary and dolphin *TRGC* genes) represent the two major paraphyletic groups. Nodes 1–4 are described in the text. The IMGT 6-letter for species (Susscr, Bostau, Oviari, Caphir, Camdro, Turtru, and Homsap) standardized abbreviation for a taxon is used.

In detail, the origin of the *TRGC* genes within the Cetartiodactyla superorder seems to have started from a common ancestral gene, which would have duplicated approximately 70 Mya ago, as indicated by node 1, which resolves the cetartiodactyl *TRGC* genes in two principal paraphyletic groups (A and B in Figure 4).

In A, only ruminant and pig *TRGC* genes were present. Therefore, the first TRGC duplicative event would have occurred only in Suina and Ruminantia lineages and, consequently, after Tylopoda and Cetacea divergence. Differently, the most evolutionarily ancient *TRGC* gene (the *TRGC5* pig-related gene) is still maintained across all the species, including dromedaries and dolphins, as indicated by the monophyletic branch B. In fact, it groups the pig, ruminant, and camel *TRGC5* and the dolphin *TRGC* genes. It is evident that strong selective pressure has maintained this ancient gene in the different cetartiodactyl genomes for a long period of evolutionary time. The high homology of this gene in the different species, which conserves clear orthology, is certainly related to a functional constraint of its protein product.

Furthermore, different from the dolphin *TRGC*, which remained unique during evolution, the dromedary *TRGC5* gene gave rise, approximately 50 Mya, to the birth of the *TRGC1* and *TRGC2* genes as indicated by the species-specific clustering (node 3 in Figure 4). The evolution of the dromedary *TRGC* genes, as a group apart from the other Cetartiodactyla suborders, would support phylogenomic research about an early divergence of Tylopoda [8,44].

Likewise, in branch A, the ruminant and pig *TRGC* genes formed distinct clades. They originated from the duplicated ancestral gene (the *TRGC6* pig-related gene) that would have duplicated itself approximately 63 Mya, after Suina and Ruminantia speciation (node 2 in Figure 4). Therefore, subsequent duplication events gave rise to the *TRGC3* and *TRGC4* genes within pig lineage distinctly from the *TRGC1*, *TRGC2*, *TRGC3*, *TRGC4*, and *TRGC6* genes generated in the ruminant lineage (Figure 4). All the ruminant *TRGC* genes appear to have evolved from a common ancestor, as they maintain clear orthology between sheep, goats, and cattle, except for the *TRGC1* and *TRGC2* genes, for which the evolutionary duplications seem to have occurred independently within Bovinae and Caprinae subfamilies (node 4 in Figure 4).

**Figure 5:**
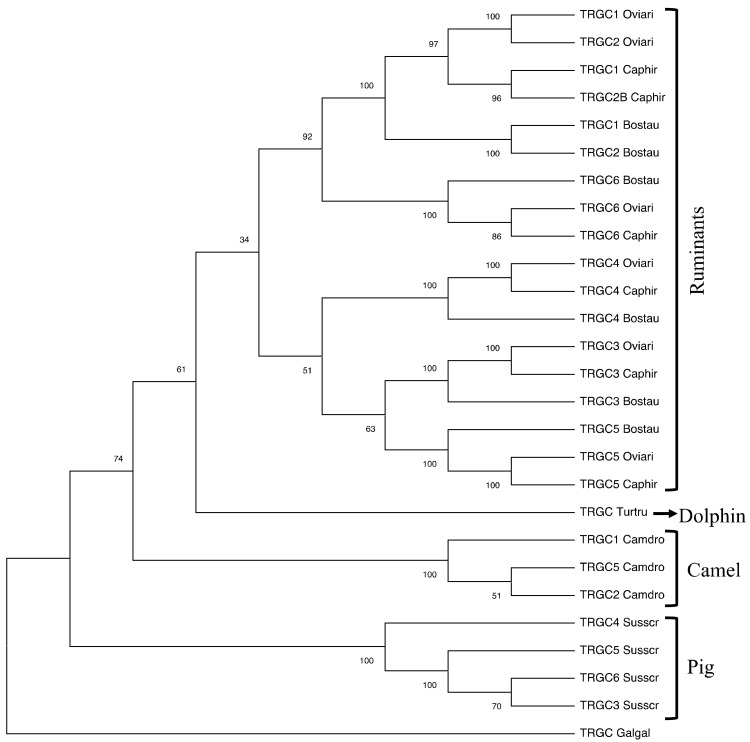
The NJ tree inferred from the pig, sheep, goat, bovine, dromedary, and dolphin 3′UTR nucleotide sequences of the *TRGC* genes. The evolutionary analyses were conducted in MEGA X [25,26]. The optimal tree with the sum of branch length = 1.76269382 is shown. The percentage of replicate trees in which the associated taxa clustered together in the bootstrap test (100 replicates) are shown next to the branches [37]. The tree is drawn to scale, with branch lengths in the same units as those of the evolutionary distances used to infer the phylogenetic tree. The evolutionary distances were computed using the p-distance method [28] and are in the units of the number of base differences per site. This analysis involved 27 nucleotide sequences. Codon positions included were 1st + 2nd + 3rd + Noncoding. All ambiguous positions were removed for each sequence pair (pairwise deletion option). There were a total of 727 positions in the final dataset. The brackets highlight lineage-specific monophyletic branches. The IMGT 6-letter for species (Susscr, Bostau, Oviari, Caphir, Camdro, Turtru, and Homsap) standardized abbreviation for a taxon is used.

We also investigated the evolutionary behavior of the flanking region of the *TRGC* genes with respect to their coding regions. The 3′UTR nucleotide sequences of the *TRGC* genes were aligned and a phylogenetic tree was constructed using the NJ method (Figure 5). The results were consistent with the previous ones. The tree perfectly recapitulated the phylogeny of the Cetartiodactyla species, grouping the sequences in lineage-specific monophyletic branches. In contrast to the coding sequences whose homology between species is preserved, the evolutionary history of their flanking regions showed a divergent pattern that groups them within lineage-specific clusters. Similarly, the flanking regions of the sheep, as well as goat *TRGC1* and *TRGC2* genes, seem to have evolved in a species-specific manner (Figure 5) with respect to their coding regions that still maintain clear homology between species (Figure 4).

### 3.6. Phylogenetic Relationships of the Cetoartiodactyla TRGC Cassettes

**Figure 6:**
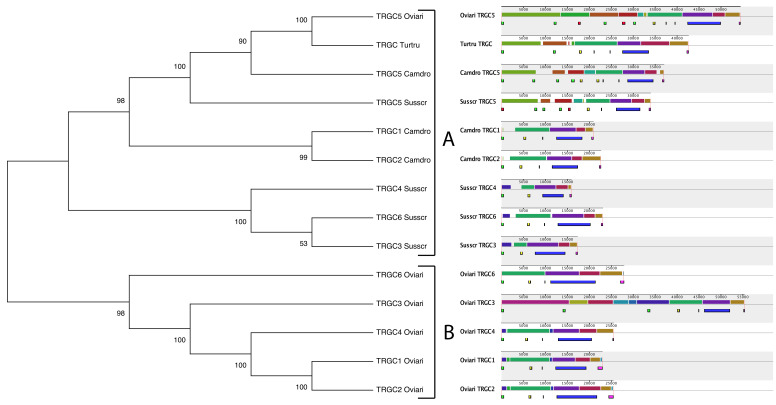
Comparison of Cetartiodactyla TRGC cassettes. Left, the NJ tree inferred from the TRGC cassette sequences retrieved from pig, sheep, dromedary, and dolphin TRG loci. The evolutionary analyses were conducted in MEGA X [25,26]. The optimal tree with the sum of branch length = 1.91796264 is shown. The percentage of replicate trees in which the associated taxa clustered together in the bootstrap test (100 replicates) are shown next to the branches [37]. The evolutionary distances were computed using the p-distance method [28] and are in the units of the number of base differences per site. This analysis involved 15 nucleotide sequences. Codon positions included were 1st + 2nd + 3rd + Noncoding. All ambiguous positions were removed for each sequence pair (pairwise deletion option). There were a total of 127,184 positions in the final dataset. Right, schematic representation of the nucleotide comparison of the same genomic region. Colored blocks highlight syntenic regions between the different sequences. Blocks below the line indicate the position of TRGV (green, yellow, and red for functional, ORF, and pseudogenes, respectively), PJ-TRGJ genes (black line), *TRGC* genes (blue), and En elements (pink) are reported. The black arrows are described in the text.

We completed our genomic analysis by comparing the entire sequences of all the TRGC cassettes retrieved within each TRG locus from different cetartiodactyl species. Each sequence starts from the ATG codon of the first *TRGV* gene located at the 5′ end of the cassette and ends with the enhancer-like element at the 3′ end. Only one representative species per Cetartiodactyla suborders was selected. The cassettes’ nucleotide sequences for the selected pig, sheep, dromedary, and dolphin TRG loci were first combined in the same alignment, and an unrooted phylogenetic tree was constructed using the NJ method [27] (left part of Figure 6).

The tree resolved the sequences into two main branches (A and B). Generally, all the cassette sequences group in a species-specific manner. The only exceptions are the pig, sheep, and dromedary TRGC5 cassettes and the dolphin TRGC single one, which form a monophyletic group within branch A, highlighting how the high level of conservation during the evolution of the different lineages affected not only the *TRGC* (Figure 4) and the *TRGV* (Figure 2) genes present in those cassettes but the entire genomic sequences of the same cassettes. This confirms, once again, the ancient origin of these cassettes.

Similar to the relative *TRGC* genes (Figure 4), the dromedary TRGC1 and TRGC2 cassettes were closely related to this conserved cassette group. Furthermore, in branch A, the pig cassette sequences formed a sister group with Tylopoda and the TRGC5 cassette group, while the relative *TRGC* genes seemed to be more related to Ruminantia genes (Figure 4). Instead, the Ruminantia cassettes sequences are grouped in a separated branch (branch B in Figure 6).

The phylogenetic analysis of the TRGC cassettes was in accordance with an overall visual inspection of their sequences (right part of Figure 6). The TRGC cassette sequences were multi-aligned for an overview, by a computational method, of the homologous regions, which are highlighted by blocks of identical colors.

Blocks with identical colors were more evident between phylogenetically related cassettes, where even the order of the homologous blocks was conserved along the sequences. As an example, the pig TRGC cassettes (except for the TRGC5) that formed a monophyletic group in the tree (branch A) were depicted by an identical string of blocks, even if with different sizes, i.e., blue, emerald, purple, brown, and dark yellow. Similarly, the ancient cassettes (pig, sheep, and dromedary TRGC5 and dolphin TRGC) shared homology regions, as the green block located at the 5′ end of each sequence. The differences were also easily detectable as the presence of an additional emerald block within the sheep TRGC5 cassette and/or the diverse size of shared blocks due to the duplication events that occurred during the sequence evolution of each species. In this context, the sheep TRGC3 cassette sequence was depicted by four colored blocks not shared with any other sequence.

However, conserved blocks of homology were shared across all the cassettes, as the purple block containing the *TRGC* genes, the emerald block representative of the *TRGV* genes, the dark yellow block located at the end of each sequence, etc., showing that evolutionary pressure to preserve the general structure of the cassettes exists.

## 4. Discussion

Among the elements of vertebrate adaptive immunity, γδ T cells represent an enigmatic population of immune cells whose role is yet to be fully elucidated. However, their unique features in γδ “high species” make animals, such as pigs, valuable models for broadening our understanding of γδ T-cell biology and for investigating the evolution of immune capacities.

In this context, the availability of a long-read pig genome assembly provided us with the opportunity to analyze, from an evolutionary point of view, the *TRG* genes within the Cetartiodactyla superorder.

First, using the current reference genome Sscrofa 11.1, we determined the genomic organization and gene content of the pig TRG locus.

During the preparation of this work, a publication became available that includes the annotation of the porcine TRG locus in three different genomic assemblies [39]. One of these was the current Sscrofa11.1 analyzed in our work. Nevertheless, we considered it appropriate to include, in this article, our annotation data deduced from Sscrofa11.1 because they were established in accordance with the ruminant gene names and the rules of the IMGT nomenclature (IMGT^®^, http://www.imgt.org; accessed on 20 December 2021). This allowed us to perform comparative analyses more easily with the other species sequences, mostly retrieved from the international IMGT database.

The pig TRG structure reflects the peculiarity of the corresponding cetartiodactyls’ loci, organized in V-J-(J)-C cassettes [13,17]. The dolphin locus represents the simplest one, with the *TRG* genes arranged in a single cassette, while the pig TRG locus, organized in four V-J-(J)-C cassettes, is in between the ruminant (six or seven cassettes) and camelid (three cassettes) loci for the number of cassettes.

The pig cassettes were classified based on the phylogenetic relationships among the *TRGV* genes and named as the ruminant corresponding cassettes. This is in accordance with the phylogenetic behavior of all the cetartiodactyl *TRGV* genes, which are grouped on the basis of their belonging to a given cassette. This allowed us to attribute the nomenclature to each pig gene and to observe that, among all, the *TRGV* genes belonging to the TRGC5 cassette conserved clearer orthology with the corresponding genes of all the species, including dolphins (Figure 2). Likewise, the *TRGC5* is the only pig *TRGC* gene to maintain conservation among the species, as indicated by the monophyletic group of branch B (Figure 4). Indeed, besides the genes, the entire sequence of the pig TRGC5 cassette, located at the 5′ end of the TRG locus, preserves extensive blocks of homology and a tight phylogenetic relationship with the corresponding one in all the species (Figure 6). As a result, the TRGC5 was the only cassette that still retained a marked homology with the corresponding TRGC cassettes of all the cetartiodactyl species analyzed in this study. Supplementary Table S2 summarizes the correspondence of the *TRGV*, the *TRGC* gene as well as the TRGC cassettes between the different species, as deduced from all phylogenetic analyses.

Therefore, our data confirmed that the pig TRGC5 cassette resembled the ancestral cassette that arose before Cetartiodactyla divergence. Its preservation for such a long evolutionary time, implies a highly specialized function of its genes, which could be essential for the survival of the species. For instance, it has been shown that the genes within the TRGC5 cassette would preferentially be expressed in specific γδ cell populations in pigs as well as in ruminants [39,45].

The functional constraint involving the coding portion of the corresponding *TRGC5* genes of the different species does not involve the non-coding regions (3′UTR) of the same genes, which instead show intraspecies conservation.

During the artiodactyls’ evolution, the ancient TRGC5 cassette underwent the first duplicative event that gave rise to the birth of the TRG loci in the different species. Each locus, consisting of reiterated cassettes, was then generated in a lineage-specific mode, as indicated by the three phylogenetic trees.

According to the time tree, the duplication involving the ancestral *TRGC5* gene would have been shared by Suina and Ruminantia, supporting Tylopoda as the first lineage to diverge [7,8]. However, the evolutionary relationships of the 3′UTRs of the *TRGC* genes show an early divergence of Suina, while Tylopoda precedes the appearance of Cetacea + Ruminantia (Cetruminantia), which is consistent with mtDNA data [11]. This information is also consistent with the phylogenetic relationships of the sequences relative to the entire TRGC5 corresponding cassettes, which form a monophyletic group within branch A (Figure 6). Nevertheless, if one observes the other TRGC cassettes, this latter tree shows a closer relationship between Suina and Tylopoda than with Ruminantia.

Overall, these data seem to confirm a certain instability at the root of the phylogeny of Cetartiodactyla, in line with previously reported results. Certainly, the different results could be related to differences in phylogenetic inference methods, molecular markers, taxon sampling, and outgroup choice. It is also clear that, despite immense progress in recent years, phylogenetic reconstruction involves many challenges that create uncertainty with respect to the true historical relationships of the organisms or genes analyzed. In fact, given the role of the immune-receptor genes linked to the habits of the life of each species, our molecular markers might not be so appropriate for completely solving controversial relative to the phylogeny of the Cetartiodactyla superorder. Anyway, our analysis highlighted the existence of a closer relationship between Suina and Tylopoda with respect to the Cetacea and Ruminantia clades. Indeed, the common data obtained from all the phylogenetic trees is that Suina and Tyolopoda branches are always tightly related although the first lineage to diverge alternates between Suina or Tylopoda.

## 5. Conclusions

In conclusion, our study, with the analysis of the pig *TRG* genes, contributes to the knowledge of the evolution of the TRG locus within Cetartiodactyla, confirming that this TR locus is the most considerably different across vertebrates and seems to be related to the evolution of species. The great plasticity supports the need to investigate the TRG genomic organization in as many species as possible to provide insight into the unique features of the T cells.

## Data Availability

The data presented in this study are available within the article or Supplementary Materials.

## Supplementary Materials

The following are available online at https://www.mdpi.com/article/10.3390/genes13020177/s1, Figure S1: (A) Structure of the pig *TRGV* genes based on the IMGT Protein display. The deduced amino acid sequences of the *TRGV* genes were manually aligned according to IMGT unique numbering for the V-REGION [35] to maximize homology. Only the functional genes and the in-frame *TRGV4* pseudogene are shown. All sequences exhibited the typical framework regions (FR) and complementarity determining regions (CDR) and the four amino acids (depicted and indicated in bold): cysteine 23 (1st-CYS) in FR1-IMGT, tryptophan 41 (CONSERVEDTRP) in FR2-IMGT, hydrophobic (here L and F) 89, and cysteine 104 (2nd-CYS) in FR3-IMGT, with the exception of the *TRGV12-2* gene that lacked the CONSERVED-TRP. Conversely, CDR-IMGT varied in amino acid composition and length. In particular, the CDR1-IMGT was five, eight, or nine amino acid lengths (AA) long. The CDR2-IMGT ranged in length from four to eight AA and the germline CDR3-IMGT was four or five AA long. Conversely, FR-IMGT varied above all in amino acid composition. The description of the strands and loops and the FR-IMGT and CDR-IMGT was according to the IMGT unique numbering for V-REGION [35]. The amino acid length of the CDR-IMGT is also indicated in square brackets. (B) Nucleotide and deduced amino acid sequences of the pig *TRGJ* genes. The consensus sequence of the heptamer and nonamer is provided at the top of the figure and is underlined. The numbering adopted for the gene classification is reported on the left of each gene. The donor splice site for each *TRGJ* is shown. The canonical FGXG amino acid motifs are underlined. Nonfunctional *TRGJ* genes are indicated in italics. (C) IMGT Protein display of the pig *TRGC* genes. The descriptions of the strands and loops were collected according to the IMGT unique numbering for the C-DOMAIN [46]. The extracellular region (C domain) is shown with black letters, the connecting region is in orange, the trans-membrane region is in purple, and the cytoplasmic region is in pink. 1st-CYS C23, CONSERVED-TRP W41, and hydrophobic AA L89 and 2nd-CYS C104 are colored (IMGT color menu) and in bold, Figure S2: Protein display of cDNA clones derived from the 5′ RACE assay (clones series GL) and IMGT database (clones series AB) in comparison with the corresponding germline *TRGV* genes. The *TRGV*, *TRGJ*, and *TRGC* gene names are listed, respectively, at the left and right of the figure. Leader region (L-REGION), CDR-IMGT, and FR-IMGT are indicated according to the IMGT unique numbering for V-DOMAIN. The four amino acids, Cysteine 23 (1st-CYS) in FR1-IMGT, tryptophan 41 (CONSERVED-TRP) in FR2-IMGT, hydrophobic (here L and F) 89, and cysteine 104 (2nd-CYS) in FR3-IMGT, with the exception of *TRGV12-2* gene that lacked the CONSERVED-TRP, are highlighted. The name of the clones is also reported. The amino acid change is boxed, Figure S3: Comparison of cis-control elements in the human and pig *TRG* loci. The nucleotide sequences of the human and pig PJγ germline promoters (A), and enhancers (B) were aligned. In (A), the alignment of conserved 400 bp between human and pig PJγ germline promoters is shown. Two conserved motifs for STAT5 proteins, one typical (TTCNNNGAA) and a second atypical (TTCNNNGTA) at 5 bp downstream of the first motif are shown by red boxes. The J-RS sequences are also boxed. In (B), the alignment of conserved 153 bp between human and pig enhancers is shown. The three (NFγ2-NFγ4) nuclear protein binding sites protected from DNase I digestion [47], are underlined. NFγ2-NFγ4 constitute the minimal enhancer fragment. NFγ2 conserves the STAT5 motif, while in NFγ3, the most extensively protected site from DNase I digestion, the conserved PEBP2 transcription factor site was found. The position of the promoter and enhancer regions in the pig TRG locus are reported in Supplementary Table S1, Table S1: Description of the related and no related TRG genes in the *Sus scrofa* chromosome 9 genome assembly (NCBI Reference Sequence NC_010451.4). The position of all genes and their classification and functionality are reported. The position of the J promoters (PJ) and enhancer elements (EnC) are also inserted, Table S2: Correspondence of the *TRGV* and *TRGC* genes, and the TRGC cassettes between the different species as deduced from the phylogenetic analysis.
