# Supplementary material for: The Organization of the Pig T-Cell Receptor γ (TRG) Locus Provides Insights into the Evolutionary Patterns of the TRG Genes across Cetartiodactyla
Source: Genes (Basel). 2022 Jan 19;13(2):177. doi: 10.3390/genes13020177 (PMC8872565; doi:10.3390/genes13020177)
Supplement: Supplementary file 1 [file genes-13-00177-s001.zip › genes-1543383-supplementary/Supplementary Files/Figure S1.pdf]

(A)

Gene	Functionality	LEADER	FR1-IMGT (1-26) A (1-15) 1 10 15	B (16-26) 16 23 26	CDR1-IMGT (27-38) BC (27-38) 27 38	FR2-IMGT (39-55) C (39-46) 39 41 46	C' (47-55) 47 55	CDR2-IMGT (56-65) C'C" (56-65) 56 65	FR3-IMGT (66-104) C" (66-74) 66 74	D (75-84) 75 80 84	E (85-96) 85 89 96	F (97-104) 97 104	CDR3-IMGT (105-117) FG (105-117) 105	
TRGV3	F	MSVLEAFTFLSFWALGLS	LSRVEQSQISVSAEV	KKSVDIN**C**KIE	STNF....PTEA	VH**W**YRQRM	NQALEHLIF	VSSV..TVPA	QSQIGGKSN	KVVARKNSQT	FTST**L**TVNFIEK	EDEGIYY**C**	AGWN...	[8.8.4]
TRGV4	P	MWIFLVSLAWVYGET	KMRISQDQLSST*RL	DRTVQIS**C**KLS	GVPL....KNAI	VH**W**YQQKE	GEPLK*ILY	GS......TN	SYQLDEPNS	SLETVTK.DK	GIFY**L**VISNVVK	SDEATYY**C**	ACWD...	[8.4.4]
TRGV6	F	MLGCLALLWALLVPGA	QEIRLVQLPVIVSRV	GNSVTMP**C**RIN	TSV.......SY	IH**W**YRQLE	GQAPERLLY	LATS..QRDV	QWDSVLQGN	KVTAKKGNDD	KSCT**L**SLMKLEK	SDEGLYY**C**	AAWDS..	[5.8.5]
TRGV7	F	MALLEAVVFSFFWAVGLG	MLRLEQPEISITAAR	QKSALIA**C**KVS	SENF....NDAY	IH**W**YRHKS	DQGMEHLMY	HVT....VFT	LSHLGEKKN	KLEARKIAST	STAT**L**KINFLEQ	EDEATYY**C**	ASWFY..	[8.6.5]
TRGV10	F	MVIQTRLTNFAIALNLSGRQYTSG	DILTTHPVASITKKR	GNTAFLE**C**QIK	TDTLK...RNVY	TH**W**YRQKP	DQPLKRILY	ISSN...ENI	VYEQGISEE	KYEARKWLSN	SLVN**L**RIHGVTE	EDTGLYY**C**	ACWN...	[9.7.4]
TRGV12-1	F	MRFPGVLLVFLAPVTQV	SSNVEGDKMSVTRAT	GSSLVIT**C**DLT	QNT.......NY	IH**W**YRFRE	GTVPQRLLY	YDVS..SSKV	TLESGISPG	RYHAYQS.AG	KSYQ**F**VISNLEE	SDSGTYY**C**	AVWEK..	[5.8.5]
TRGV12-2	F	MRFPGVLLVFLVPVTQV	SSNVEGDKMSVTRAP	GTAVSFT**C**ALT	QNT.......NY	IH**V**YTKKE	GTAPQRLFY	YDIY..YSKF	TLESGDSPE	KYRVYAG.TS	KSYT**F**TMLYLEE	RDSGTYY**C**	ALWDK..	[5.8.5]

(B)

TRGJ gene name	J-NONAMER GGTTTTTGT	J-SPACER ************	J-HEPTAMER CACTGTG	J-REGION	5'splice donor
TRGJ3-1	gtttttttgt	aggagctttaac	cagtgtg	ATAACTGGAAGAAGATATTTGGAAAGGCACTGGAGCTCATAGTAGCTCCCTATG<br>N W K K I F G K A L E L I V A P Y	gtaagt
TRGJ4-1	*gtttttttgt*	*aggagctttaac*	*cagtgtg*	*ATAAACTGGAAGAAGATATTTGAAAAGGCACTGGAGCTCATAGTAGCTCCCTATG*<br>*I N W K K I F E K A L E L I V A P Y*	*gtaagt*
TRGJ5-1	ggtttttat	aggagctttaac	cagtgtg	ATAGCTCACGCTGGATCAAGATATTTGGAGAGGGGACTAAGCTCATAGTAACTCCCCCTG<br>S S R W I K I F G E G T K L I V T P P	gtaagt
TRGJ5-2	agtttttga	tatagattgaat	cactgtg	GCCTGATTACGTAAAAGTCTTCGGAGATGGAACAAAACTTGCTGTTACA<br>P D Y V K V F G D G T K L A V T	gtaggt
TRGJ6-1	*ggaatttttt*	*gtaggcactcta*	*agcagtg*	*ATAACTCAGGCTGGATCAAGATATTTGGAGAAGGGAGTAAACTCATAGAAACTCCCCCTG*<br>*N S G W I K I F G E G S K L I E T P P*	*gtaagt*
TRGJ6-2	agtttttga	tatgggttgaat	cactgtg	GACTTCATACAACAAAATCTTCGGTGCTGGAACAAAACTTTTCGTCATAG<br>T S Y N K I F G A G T K L F V I	gtaggt

(C)

```
                                                                         [EX1]
                       A          AB       B            BC          C      CD       D           DE             E       EF   F          FG          G
                     (1-15)             (16-26)      (27-38)     (39-45)        (77-84)                     (85-96)      (97-104)  (105-117)   (118-128)
                 ---------------->  ----------->                 ------->       ------->                ------------->  ---------->              -----------
                   1         10   15   16     23  26 27       38 3941  45        77    84                       85   89     96  97    104 105       117 118
                87654321|........|....|123|......|..| |........| |.|...|1234567|......|12345677654321|...|......|12|......| |...........| |........
Susscr TRGC3   (D)INLASDISPKPTVFLPSIAEIKL..HNAGTYLCLLE NFFP..NVIK VYWKEKNGNKVL..ESQQGNTMRTTN.......TYMKFSWLTVSKT....AMDKEHKC VVKHEKNRGGVDQ EIIFPSVNE
Susscr TRGC4   (D)INLASDLSPKPTVFLPSIAEIKL..HNAGTYLCLLE NFFP..NVIK VYWKEKNGNKVL..ESQQGNTMRTTN.......TYMKFSWLTVSKT....AMDKEHKC VVKHEKNRGGVDQ EIIFPSVNE
Susscr TRGC5   (D)RNLAADMSPKPTIFLPSIAEINL..HNTGTHLCLLE KFFP..DAIK VYWKEKNGNTVL..DSQQGDVIQTND.......TYMKFSWLTVTQK....SADKEHIC IVKHENNKRGTDQ EILFPSMNK
Susscr TRGC6   (D)RNLATDMTPKPTVFLPSIAEIEH..SKVGTYLCLLE KFFP..NAIQ VYWKEKNDNRVL..ESMQGNTVKTDD.......TYMKFSWLTVSEE....SMGKEHIC FVKHEKNRVEPVQ EILFPSINE
```

```
                                   CONNECTING-REGION                                                             TRANSMEMBRANE-REGION     CYTOPLASMIC-REGION
                    [EX2A]                                [EX2B]               [EX2C]                                     [EX3]
Susscr TRGC3    (V)VSSAVMPTDSPNDCLKDES              (K)VSDSDSRKACVRNES   (E)VTNSTKACLKDES      (N)TLQLQLENTSAYYTYLLLLLKSSLYFATTISCLFRRTV.CSSGKTS
Susscr TRGC4    (V)VTSVVTTTEPPNDCLNDGS              (K)ATGIDSEKACKKDHS   (E)VTVTDSKKVCQKDES    (N)SLQLQLENTSAYYTYLLLLLKSTLYFAIITCCLFRRTV.CSSGKTS
Susscr TRGC5    (E)VTTDVCLKDTS                                                                 (G)TMQLQLTNTSAYYTYLLLLLKSALYFAIITYCLFRETTVCSAWKRS
Susscr TRGC6    (V)VSSAVMPTDSPNDCLKDES              (K)VSDSDSRKACVRNES   (E)ITNSTKACLKDES      (N)TLQLQLENTSAYYTYLLLLLKSTLYFATVLSCLFRRTV.CSSGKTS
```
