# Supplementary material for: The Organization of the Pig T-Cell Receptor γ (TRG) Locus Provides Insights into the Evolutionary Patterns of the TRG Genes across Cetartiodactyla
Source: Genes (Basel). 2022 Jan 19;13(2):177. doi: 10.3390/genes13020177 (PMC8872565; doi:10.3390/genes13020177)
Supplement: Supplementary file 1 [file genes-13-00177-s001.zip › genes-1543383-supplementary/Supplementary Files/Figure S2.pdf]

```
                                                      FR1-IMGT         CDR1-IMGT        FR2-IMGT      CDR2-IMGT                   FR3-IMGT                     CDR3-IMGT        FR4-IMGT
                                                       (1-26)           (27-38)          (39-55)       (56-65)                    (66-104)                     (105-117)        (118-128)
                                                  A              B         BC           C         C'        C'C"           C"         D           E          F             FG                G
                                                (1-15)        (16-26)   (27-38)    (39-46)   (47-55)    (56-65)     (66-74)   (75-84)    (85-96)    (97-104)       (105-117)         (118-128)
TRGV    Clone                             --------------> ----------->              -------> -------->            --------> --------> -----------> -------->                       ----------->   TRGJ   TRGC
gene     name                L-REGION     1        10  15 16    23 26 27         38 3941  46 47      55 56      65 66     74 75   80 84 85  89    96 97    104 105 111      112 117 118      128   gene   gene
                                          |........|....| |......|..| |..........| |.|....| |.......| |........| |.......| |....|...| |...|......| |......| |.....|1234321|....| |.........|
TRGV6                    MLGCLALLWALLVPGA QEIRLVQLPVIVSRV GNSVTMPCRIN TSV.......SY IHWYRQLE GQAPERLLY LATS..QRDV QWDSVLQGN KVTAKKGNDD KSCTLSLMKLEK SDEGLYYC AAWDS
        GL2L6            -------------------------------- GNSVTMPCRIT TSV.......SY IHWYRQLE GQAPERLLY LATS..QRDV QWDSVLQGN KVTAKKGNDD KSCTLSLMKLEK SDEGLYYC AAWDSNPPP......SYNKI FGAGTKLFVI..   6-2     C6
        GL2L13           -------------------------------- GNSVTMPCRIT TSV.......SY IHWYRQLE GQAPERLLY LATS..QRDV QWDSVLQGN KVTAKKGNDD KSCTLSLMKLEK SDEGLYYC AAWDSDTPP......SYNKI FGAGTKLFVI..   6-2     C6
        AB185445         MLGCLALLWALLVPGH QEIRLVQLPVIVSRV GNSVTMPCRIT TSV.......SY IHWYRQLE GQAPERLLY LATS..QRDV QWDSVLQGN KVTAKKGNDD KSCTLSLMKLEK SDEGLYYC AAWDSVE........SYNKI FGAGTKLFVI..   6-2     C6


TRGV12-1                MRFPGVLLVFLAPVTQV SSNVEGDKMSVTRAT GSSLVITCDLT QNT.......NY IHWYRFRE GTVPQRLLY YDVS..SSKV TLESGISPG RYHAYQS.AG KSYQFVISNLEE SDSGTYYC AVWEK
        GL2L17          ------LLVFLAPVTQV SSNVEGDKMSVTRAT GSSLVITCDLT QNT.......NY IHWYRFRE GTVPQRLLY YDVS..SSKV TLESGISPG RYHAYQS.AG KSYQFVISNLEE SDSGTYYC AVWE...............I FGKALELIVAPY   3-1     C3
        GL2L36          MRFPGVLLVFLAPVTQV SSNVEGDKMSVTRAT GSSLVITCDLT QNT.......NY IHWYRFRE GTVPQRLLY YDVS..SSKV TLESGISPG RYHAYQS.AG KSYQFVISNLEE SDSGTYYC AVWEKL.........NWKKI FGKALELIVAPY   3-1     C3
        AB185446        MRFPGVLLVFLAPVTQV SSNVEGDKMSVTRAT GSSLVITCDLT QNT.......NY IHWYRFRE GTVPQRLLY YDVS..SSKV TLESGISPG RYHAYQS.AG KSYQFVISNLEE SDSGTYYC AAP............NWKKI FGKALELIVAPY   3-1     C3


TRGV12-2                MRFPGVLLVFLVPVTQV SSNVEGDKMSVTRAP GTAVSFTCALT QNT.......NY IHVYTKKE GTAPQRLFY YDIY..YSKF TLESGDSPE KYRVYAG.TS KSYTFTMLYLEE RDSGTYYC ALWDK
        AB185447        MRFPGVLLVFLVPVTQV SSNVEGDKMSVTRAP GTAVSFTCALT QNT.......NY IHVYTKKE GTAPQRLFY YDIY..YSKF TLESGDSPE KYRVYAG.TS KSYTFTMLYLEE RDSGTYYC ALWGY...........WKKI FEKALELIVAPY   4-1     C4


TRGV3                  MSVLEAFTFLSFWALGLS LSRVEQSQISVSAEV KKSVDINCKIE STNF....PTEA VHWYRQRM NQALEHLIF VSSV..TVPA QSQIGGKSN KVVARKNSQT FTSTLTVNFIEK EDEGIYYC AGWN
        GL1L2          -------------------------------------------------NF....PTEA VHWYRQRT NQALEHLIF VSSV..TVPA QSQIGGKSN KVVARKNSQT FTSTLTVNFIEK EDEGIYYC AGWVNY.......SSRWIKI FGEGTKLIVTPP   5-1     C5


TRGV7                  MALLEAVVFSFFWAVGLG MLRLEQPEISITAAR QKSALIACKVS SENF....NDAY IHWYRHKS DQGMEHLMY HVT....VFT LSHLGEKKN KLEARKIAST STATLKINFLEQ EDEATYYC ASWFY
        AB185441       MALLEAVVFSFFWAVGLG MLRLEQPEISITAAR QKSALIACKVS SENF....NDAY IHWYRHKS DQGMEHLMY HVT....VFT LSHLGEKKN KLEARKIAST STATLKINFLEQ EDEATYYC ASWFYSFY........YVKV FGDGTKLAVT..   5-2     C5
        AB185442       MALLEAVVFSFFWAVGLG MLRLEQPEISITAAR QKSALIACKVS SENF....NDAY IHWYRHKS DQGMEHLMY HVT....VFT LSHLGEKKN KLEARKIAST STATLKINFLEQ EDEATYYC ASWFK............... .GEGTKLIVTPP   5-1     C5


TRGV10           MVIQTRLTNFAIALNLSGRQYTSG DILTTHPVASITKKR GNTAFLECQIK TDTLK...RNVY THWYRQKP DQPLKRILY ISSN...ENI VYEQGISEE KYEARKWLSN SLVNLRIHGVTE EDTGLYYC ACWN
        AB185443 MVIQTRLTNFAIALNLSGRQYTSG DILTTHPVASITKKR GNTAFLECQIK TDTLK...RNVY THWYRQKP DQPLKRILY ISSN...ENI VYEQGISEE KYEARKWLSN SLVNLRIHGVTE EDTGLYYC ACWNI..........DYVKV FGDGTKLAVT..   5-2     C5


TRGV4                       MWIFLVSLAWVYGET KMRISQDQLSST*RL DRTVQISCKLS GVPL....KNAI VHWYQQKE GEPLK*ILY GS......TN SYQLDEPNS SLETVTK.DK GIFYLVISNVVK SDEATYYC ACWD
        AB185444            MWIFLVSLAWVYGET KMRISQDQLSST*RL DRTVQISCKLS GVPL....KNAI VHWYQQKE GEPLK*ILY GS......TN SYQLDEPNS SLETVTK.DK GIFYLVISNVVK SDEATYYC AGRRFG.............  .GDGTKLAVT..   5-2     C5
```
