# Supplementary material for: The Organization of the Pig T-Cell Receptor γ (TRG) Locus Provides Insights into the Evolutionary Patterns of the TRG Genes across Cetartiodactyla
Source: Genes (Basel). 2022 Jan 19;13(2):177. doi: 10.3390/genes13020177 (PMC8872565; doi:10.3390/genes13020177)
Supplement: Supplementary file 1 [file genes-13-00177-s001.zip › genes-1543383-supplementary/Supplementary Files/Supplementary Table S1.pdf]

Table S1. Description of the related and no related TRG genes in the *Sus scrofa* chromosome 9 genome assembly (NCBI Reference Sequence NC_010451.4). The position of all genes and their classification and functionality are reported. The position of the J promoters (PJ) and enhancer elements (EnC) are also inserted.

Gene classification	Functionality	Position[a] (complement)
AMPH	F	108800898-108971498
TRGV11	P[1]	108791329-108791795
TRGV3	F	108783758-108784225
TRGV7	F	108781929-108782395
TRGV10	F	108778140-108778618
TRGV4	P[2]	108776148-108776578
PJ5	-	108771874-108772273
TRGJ5-1	F	108771814-108771873
TRGJ5-2	F	108768979-108769027
TRGC5	F	108760275-108765636
EnC5	-	108757853-108758005
TRGV6	F[b]	108752589-108753018
PJ6	-	108746720-108747119
TRGJ6-1	ORF[3]	108746660-108746719
TRGJ6-2	F	108743190-108743239
TRGC6	F	108732805-108740151
EnC6	-	108729968-108730120
TRGV12-1	F	108719508-108719974
PJ3	-	108715278-108715677
TRGJ3-1	F	108715224-108715277
TRGC3	F	108705517-108712310
EnC3	-	108702679-108702831
TRGV12-2	F[b]	108692578-108693036
PJ4	-	108686628-108687027
TRGJ4-1	ORF[4]	108686567-108686595
TRGC4	F[c]	108678980-108683647
EnC4	-	108677153-108677305
STARD3NL	F	108654070-108673560

[a] L-PART1/ V-exon for TRGV genes; [b]F is referred to the same gene detected within productive cDNAs; [c]F is referred to the same gene detected within productive cDNAs and Sscrofa10.2 genome assembly

[1] Frameshift in L-PART1; frameshift in V-exon

[2] Stop codons in V-exon

[3] No canonical J-HEPTAMER

[4] FEXA instead of FGXA
