# Supplementary material for: The Organization of the Pig T-Cell Receptor γ (TRG) Locus Provides Insights into the Evolutionary Patterns of the TRG Genes across Cetartiodactyla
Source: Genes (Basel). 2022 Jan 19;13(2):177. doi: 10.3390/genes13020177 (PMC8872565; doi:10.3390/genes13020177)
Supplement: Supplementary file 1 [file genes-13-00177-s001.zip › genes-1543383-supplementary/Supplementary Files/Supplementary Table S2.pdf]

Table S2. Correspondence of the TRGV and TRGC genes, and the TRGC cassettes between the different species as deduced from the phylogenetic analysis

**Ruminantia**						**Suina**		**Tylopoda**		**Cetacea**	
Capra hircus		*Ovis aries*		*Bos taurus*		*Sus scrofa*		*Camelus dromedarius*		*Tursiops truncatus*	
**cassette**	**gene**	**cassette**	**gene**	**cassette**	**gene**	**cassette**	**gene**	**cassette**	**gene**	**cassette**	**gene**
TRGC5	TRGV11	TRGC5	TRGV11-1	TRGC5	-	TRGC5	TRGV11	TRGC5	TRGV11	TRGC5	TRGV1
	TRGV3-1/2		TRGV3-1/2		TRGV3-1/2		TRGV3		TRGV3		
	TRGV7		TRGV7		TRGV7-1		TRGV7		TRGV7		TRGV2
	TRGV10		TRGV10-1		TRGV10-1		TRGV10		TRGV10		-
	TRGV4		TRGV4		TRGV4-1		TRGV4		TRGV4		-
	TRGC5		TRGC5		TRGC5		TRGC5		TRGC5		TRGC
TRGC3	TRGV8	TRGC3	TRGV8-1	TRGC7	TRGV8-1/2/3/4	-	-	-	-	-	-
	TRGV2		TRGV2-1		TRGV2-1		-		-		-
	-		-		TRGC7*		-		-		-
	TRGV9		TRGV9-1	TRGC3	TRGV9-1/2	TRGC3	TRGV12-1	TRGC1	TRGV1	-	-
	TRGC3		TRGC3		TRGC3		TRGC3		TRGC1		-
TRGC4	TRGV1	TRGC4	TRGV1	TRGC4	TRGV1-1	TRGC4	TRGV12-2	-	-	-	-
	TRGC4		TRGC4		TRGC4		TRGC4		-		-
TRGC1	TRGV5-1	TRGC1	TRGV5-1	TRGC1	TRGV5-1	-	-	-	-	-	-
	TRGC1		TRGC1		TRGC1		-		-		-
TRGC2A	TRGV5-2	-		-		-	-	-	-	-	-
	TRGC2A*						-		-		-
TRGC2B		TRGC2		TRGC2	TRGV6-1	-	-	-	-	-	-
	TRGV5-3		TRGV5-2		TRGV5-2		-		-		-
	TRGC2B		TRGC2		TRGC2		-		-		-
TRGC6	TRGV6	TRGC6	TRGV6-1	TRGC6	TRGV6-2	TRGC6	TRGV6	TRGC2	TRGV2	-	-
	TRGC6		TRGC6		TRGC6		TRGC6		TRGC2		-

Legend:
- (*) indicates TRGC pseudogenes not included in the phylogenetic study;
- identical colours highlight the corresponding TRGC cassettes among species;
- the TRGV genes highlighted with a same colour derive from the groupings of Figure 2.
